# Adding Docetaxel to Tepotinib to Overcome Oligoprogression in MET Exon 14 Skipping–Mutated NSCLC When Local Therapy Is Unfeasible: A Case Report

**DOI:** 10.1155/crom/4483379

**Published:** 2025-08-19

**Authors:** David Sánchez García, Beatriz Grau Mirete, Paula Rodríguez Payá, Asia Ferrández-Arias, Miguel Borregón-Rivilla, Antonio-David Lázaro-Sánchez, Javier-David Benítez-Fuentes

**Affiliations:** ^1^Department of Medical Oncology, Hospital General Universitario de Elche, Alicante, Spain; ^2^Department of Medical Oncology, Hospital General Universitario Morales Meseguer, Murcia, Spain

**Keywords:** docetaxel, METex14 skipping, NSCLC, oligoprogression, tepotinib

## Abstract

Oligoprogression in MET Exon 14 skipping (METex14)–mutated non–small cell lung cancer (NSCLC) is clinically challenging, particularly when local therapies are contraindicated. We report the first documented case of a 62-year-old man with oligoprogressive METex14-positive NSCLC who achieved a sustained metabolic response following the addition of docetaxel to ongoing tepotinib therapy after progression on tepotinib monotherapy. Due to prior thoracic irradiation, reirradiation and surgical interventions were deemed not feasible, prompting this systemic combination to maintain MET inhibition while targeting resistant tumor clones. This strategy resulted in a partial metabolic response at the primary lung lesion and a sustained complete metabolic response in an adrenal metastasis. The regimen was generally well tolerated; however, Grade 3 peripheral edema required dose reduction of tepotinib. This case supports the potential role of systemic therapy intensification in METex14-driven NSCLC, highlighting the therapeutic value of continued MET inhibition beyond disease progression, particularly when local treatment and advanced molecular monitoring such as ctDNA are unavailable.

**Trial Registration:** ClinicalTrials.gov identifier: NCT05439993

## 1. Introduction

Non–small cell lung cancer (NSCLC) with MET Exon 14 skipping (METex14) mutations is a distinct, aggressive subtype with historically poor outcomes. Found in 3%–4% of NSCLC cases, these mutations drive oncogenic signaling via constitutive MET activation, making them targetable with MET inhibitors such as tepotinib and capmatinib [1, 2]. Although these therapies achieve objective response rates of 40%–60% and a median progression-free survival of 10–12 months in clinical trials, resistance inevitably develops [2]. Managing oligoprogression is challenging, as local therapies like radiotherapy are often preferred but may be limited by anatomical constraints or cumulative toxicity [3].

Current guidelines recommend discontinuing MET inhibitors upon progression and transitioning to chemotherapy, immunotherapy, or a combination of both [4]. However, this strategy halts MET inhibition, potentially allowing rapid regrowth of sensitive tumor clones.

Here, we present the first reported case of oligoprogressive METex14-mutated NSCLC treated with tepotinib and docetaxel after primary tumor progression on tepotinib monotherapy, where reirradiation and surgery were deemed unfeasible. This approach achieved a sustained metabolic response with manageable toxicity.

## 2. Case Report

**Figure 1:**
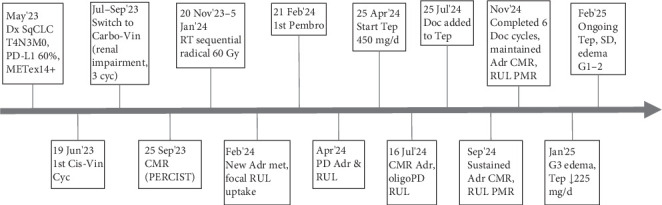
Timeline of events. Adr, adrenal; Carbo, carboplatin; Cis, cisplatin; CMR, complete metabolic response; Cyc, cycle; d, day; Doc, docetaxel; Dx, diagnosis; G, grade (toxicity grading); METex14+, MET Exon 14 skipping mutation-positive; met, metastasis; oligoPD, oligoprogression; PD, progressive disease; PD-L1, programmed death ligand-1; Pembro, pembrolizumab; PERCIST, positron emission tomography response criteria in solid tumors; PMR, partial metabolic response; RT, radiotherapy; RUL, right upper lobe; SD, stable disease; Seq, sequential; SqCLC, squamous cell lung cancer; Tep, tepotinib; Vin, vinorelbine; ↓, dose reduced.

In May 2023, a 62-year-old Caucasian male with a 30-pack-year smoking history (quit in 1991) and well-controlled dyslipidemia presented with a 2-month history of dry cough and 6 kg of unintentional weight loss. His ECOG performance status was 0. Contrast-enhanced CT revealed an 80 × 64 mm right upper lobe (RUL) mass with mediastinal invasion, along with ipsilateral hilar, subcarinal, paratracheal, and supraclavicular lymphadenopathy (short-axis: 25 mm). PET-CT showed intense metabolic activity in the primary lesion (SUVmax 18.2), mediastinal nodes (SUVmax 12.5), and right supraclavicular lymphadenopathy (SUVmax 5.57) consistent with stage T4 N3 M0 per the eighth TNM edition. A transbronchial biopsy confirmed squamous cell carcinoma. Next-generation sequencing with Oncomine Comprehensive Assay v3 identified a METex14 mutation (variant allele frequency [VAF] of 61%), detected both at DNA and RNA, with the RNA analysis demonstrating an exon-skipping event between Exons 13 and 15. No co-occurring alterations were found. PD-L1 immunohistochemistry showed a tumor proportion score (TPS) of 60%. The timeline of events is summarized in Figure 1.

The multidisciplinary thoracic malignancies tumor board recommended definitive-intent chemoradiotherapy. On June 19, 2023, the patient received the first cycle of cisplatin 75 mg/m^2^ and intravenous (iv) vinorelbine 25 mg/m^2^. However, Grade 1 renal dysfunction (creatinine: 1.8 mg/dL, per CTCAE v5.0, compared with a baseline creatinine level of 1.3 mg/dL) led to a switch to carboplatin AUC5 with iv vinorelbine 25 mg/m^2^ for three additional cycles from July to September 2023. After which creatinine levels improved until reaching a baseline creatinine value of 1.3 mg/dL. Restaging PET-CT on September 25, 2023, showed a reduction in the RUL mass size (21 × 11 mm), along with a complete metabolic response (CMR) in all lesions according to PERCIST criteria. Sequential radiotherapy (60 Gy in 30 fractions) was delivered to the primary tumor and involved lymph nodes from November 20, 2023, to January 5, 2024. The treatment was complicated by Grade 2 esophagitis, managed with supportive care and dietary modifications.

Had disease progression not occurred, consolidation therapy with durvalumab would have been initiated. However, in February 2024, surveillance PET-CT detected a new 35 × 21 mm right adrenal metastasis (SUVmax 12.1) and focal uptake in the RUL (SUVmax 6.8), initially attributed to postradiation inflammation. A brain MRI showed no radiographic evidence of intracranial metastases. Adrenal biopsy confirmed metastatic squamous cell carcinoma with the same histopathological and molecular characteristics as the original tumor, including a PD-L1 TPS of 60%. Pembrolizumab 200 mg iv every 3 weeks was initiated on February 21, 2024. After three cycles, April 2024 CT imaging demonstrated disease progression, with a significant increase in the right adrenal metastasis to 50 × 35 mm and further enlargement of the RUL mass to 34 × 21 mm according to PERCIST criteria. Immunotherapy was discontinued due to progression.

Based on the molecular profile and the availability of approved second-line targeted therapy, oral tepotinib (450 mg daily) was initiated on April 25, 2024. Follow-up PET-CT on July 16, 2024, showed a CMR of the adrenal metastasis, which was metabolically inactive and had decreased in size to 17 × 25 mm, along with evidence of oligoprogression at the primary RUL lesion, which increased to 49 × 37 mm (SUVmax 6.8). Circulating tumor DNA (ctDNA) analysis was unavailable at our institution. A CT-guided biopsy of the progressing lesion was performed, and molecular profiling was carried out using the Oncomine Precision Assay (50 genes), a targeted NGS panel different from the initial comprehensive genomic profiling performed at baseline (Oncomine Comprehensive Assay v3, 161 genes). This analysis confirmed the persistence of the original METex14 mutation (VAF 57.4%) and revealed a newly acquired amplification of the BRAF gene (Copy Number: 20.63). Due to prior thoracic radiation, reirradiation was considered high-risk because of potential dose-related toxicity to critical structures such as the esophagus and spinal cord.

Given the limited treatment options, adding docetaxel to ongoing tepotinib was requested as an off-label strategy to target resistant tumor clones while preserving systemic MET inhibition, and it was approved by the hospital and pharmacy. On July 25, 2024, docetaxel was initiated at a preemptive reduced dose (45 mg/m^2^, 60% of the standard) to minimize overlapping toxicities, particularly edema and fluid retention.

**Figure 2:**
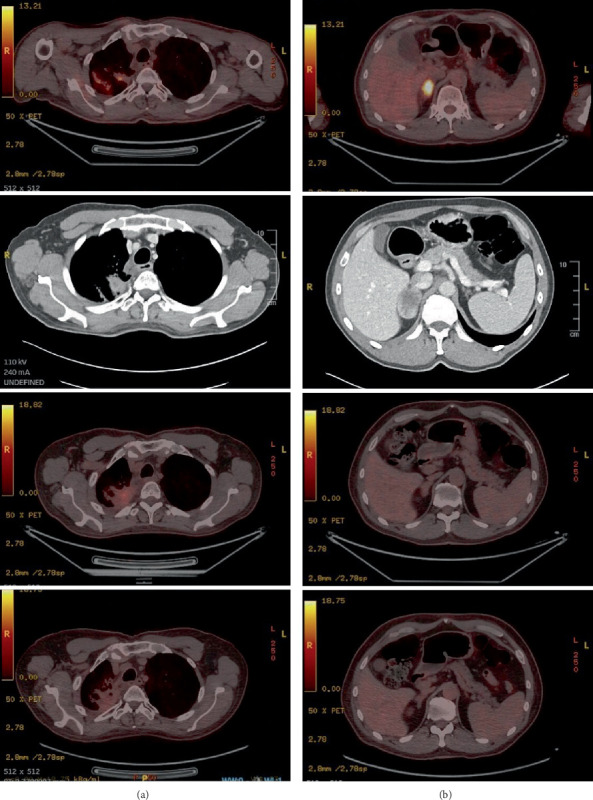
Sequential imaging demonstrating progression and therapeutic responses of the (a) primary lung lesion and the (b) adrenal metastasis across four time points. The top row (February 2024 PET-CT) shows initial progression with a newly detected adrenal metastasis. The second row (April 2024 CT) demonstrates further progression under pembrolizumab, prompting initiation of tepotinib. The third row (July 2024 PET-CT) reveals a complete metabolic response (CMR) in the adrenal lesion but oligoprogression of the lung lesion, leading to docetaxel addition. The bottom row (September 2024 PET-CT) confirms a partial metabolic response (PMR) of the lung lesion while the adrenal metastasis maintains CMR.

After three cycles, a PET-CT in September 2024 demonstrated a partial metabolic response (PMR) at the primary lung site (SUVmax 3.85) with a previous value of SUVmax 6.8 according to PERCIST criteria. The adrenal lesion remained metabolically inactive, consistent with a sustained CMR. The patient completed six cycles of docetaxel in November 2024, after which it was discontinued, maintaining a PMR in the RUL mass and sustained CMR in the adrenal, with no new lesions (Figure 2).

The combination was initially well-tolerated; however, serum creatinine increased from a baseline of 1.3–1.85 mg/dL in July 2024 before stabilizing. After the second cycle of docetaxel, the patient developed Grade 2 peripheral edema, which was effectively managed with furosemide 20 mg daily and limb elevation. In January 2025, 6 weeks after completing docetaxel, edema progressed to Grade 3, causing mobility limitations and necessitating a tepotinib dose reduction to 225 mg daily. Edema resolved within 14 days, and renal function improved, with serum creatinine stabilizing between 1.5 and 1.6 mg/dL.

As of February 2025, the patient remains on tepotinib 225 mg daily with a preserved ECOG PS of 0 and sustained disease control. The patient remains almost asymptomatic, continues full-time employment, and reports satisfaction with treatment tolerability, with peripheral edema ranging between Grades 1 and 2 as the primary adverse event.

## 3. Discussion

Oligoprogression in oncogene-driven NSCLC poses significant challenges, with tumor growth occurring at limited sites while most disease remains controlled by targeted therapy. METex14-mutated NSCLC, a rare subset of cases, raises crucial management questions [2, 5, 6]. Current guidelines recommend either discontinuing targeted therapy upon progression or using local treatments to control oligoprogression while maintaining systemic therapy [5, 6]. However, discontinuing targeted therapy may allow previously controlled MET-driven disease to resurge, especially when local therapies are not feasible. In this case, oligoprogression was confined to the primary lung lesion but could not be addressed with local therapies due to prior radiotherapy. This highlights a key treatment gap: the need to target resistant tumor sites without compromising systemic disease control.

Preclinical data suggest that MET inhibition may enhance the efficacy of cytotoxic agents through synergistic interactions [7–9]. METex14 mutations drive constitutive MET signaling, promoting proliferation, invasiveness, and apoptosis resistance [2]. By blocking aberrant MET activity, tepotinib may not only impair tumor growth but also enhance intracellular retention of chemotherapeutic agents, potentially increasing their effectiveness [8, 9]. Taxanes, as microtubule inhibitors, induce mitotic arrest and cell death across a broad tumor spectrum, including both MET-dependent and partially MET-independent cells [8–10]. Synergy could stem from tepotinib dampening efflux transporters, allowing higher intracellular concentrations of taxanes [8]. Building on this rationale, ongoing research is evaluating a similar combination of tepotinib and paclitaxel in patients with MET-amplified or MET Exon 14-altered advanced gastric and gastroesophageal junction carcinomas.

Evidence from other oncogene-driven NSCLCs (e.g., EGFR- or ALK-altered) supports continuing targeted therapy alongside chemotherapy for oligoprogression [11–14]. Our case report supports this approach; the primary lung lesion demonstrated metabolic regression following the addition of taxanes, while the adrenal metastasis remained in CMR under continued MET inhibition.

Tepotinib frequently causes peripheral edema, reported in up to 50%–60% of patients in clinical trials [1], while docetaxel can exacerbate fluid retention among other toxicities, including myelosuppression [15]. To mitigate these additive effects, in a patient with prior lines of therapy and particularly relevant in oligoprogression, with the aim of achieving effective disease control while minimizing treatment-related toxicity, a preemptive dose reduction was implemented, with docetaxel given at 60% of the standard dose. Despite this precaution, the patient developed Grade 2 edema, which progressed to Grade 3 (6 weeks after completing docetaxel), requiring a 50% tepotinib dose reduction. Yet, disease control persisted. This outcome illustrates that proactive toxicity management (including diuretics, supportive care, and dose adjustments) may enable safe administration of this combination.

Although genomic profiling confirmed the METex14 driver mutation, the absence of ctDNA testing limited the identification of other potential resistance mechanisms that could further guide treatment selection [16].

Our experience suggests that combining docetaxel with ongoing tepotinib can effectively manage oligoprogressive METex14-mutated NSCLC, particularly when local therapies are not an option. Despite the historically poor prognosis of METex14-driven disease, this case highlights the potential of systemic intensification strategies that sustain MET inhibition while targeting resistant clones. Proactive toxicity management and dose adjustments were key to maintaining treatment tolerability. When molecular diagnostics such as ctDNA analysis or tumor biopsies are unavailable or impractical, an empiric approach combining systemic therapies may be a viable alternative.

## Data Availability

All relevant data are included within the article. Further inquiries can be directed to the corresponding author.
